# Virtual, Augmented, and Mixed Reality Applications for Surgical Rehearsal, Operative Execution, and Patient Education in Spine Surgery: A Scoping Review

**DOI:** 10.3390/medicina60020332

**Published:** 2024-02-15

**Authors:** Tim Bui, Miguel A. Ruiz-Cardozo, Harsh S. Dave, Karma Barot, Michael Ryan Kann, Karan Joseph, Sofia Lopez-Alviar, Gabriel Trevino, Samuel Brehm, Alexander T. Yahanda, Camilo A Molina

**Affiliations:** 1 Department of Neurological Surgery, Washington University School of Medicine, St. Louis, MO 63110, USA; 2 University of Pittsburgh School of Medicine, Pittsburgh, PA 15261, USA

**Keywords:** augmented reality, computer-assisted spine surgery, mixed reality, resident education, spine navigation, virtual reality

## Abstract

*Background and Objectives*: Advances in virtual reality (VR), augmented reality (AR), and mixed reality (MR) technologies have resulted in their increased application across many medical specialties. VR’s main application has been for teaching and preparatory roles, while AR has been mostly used as a surgical adjunct. The objective of this study is to discuss the various applications and prospects for VR, AR, and MR specifically as they relate to spine surgery. *Materials and Methods*: A systematic review was conducted to examine the current applications of VR, AR, and MR with a focus on spine surgery. A literature search of two electronic databases (PubMed and Scopus) was conducted in accordance with the Preferred Reporting Items for Systematic Reviews and Meta-Analyses (PRISMA). The study quality was assessed using the MERSQI score for educational research studies, QUACS for cadaveric studies, and the JBI critical appraisal tools for clinical studies. *Results*: A total of 228 articles were identified in the primary literature review. Following title/abstract screening and full-text review, 46 articles were included in the review. These articles comprised nine studies performed in artificial models, nine cadaveric studies, four clinical case studies, nineteen clinical case series, one clinical case–control study, and four clinical parallel control studies. Teaching applications utilizing holographic overlays are the most intensively studied aspect of AR/VR; the most simulated surgical procedure is pedicle screw placement. *Conclusions*: VR provides a reproducible and robust medium for surgical training through surgical simulations and for patient education through various platforms. Existing AR/MR platforms enhance the accuracy and precision of spine surgeries and show promise as a surgical adjunct.

## 1. Introduction

Virtual reality (VR), augmented reality (AR), and mixed reality (MR) are similar technologies that have undergone significant advances and adoption across multiple industries including healthcare. VR may refer to a computer-generated simulation of either the real world or a manufactured digital world. The user interacts with VR as if they were in the real world, but the focus of the interaction remains on the digital environment [1]. 

AR allows the physical environment to be enhanced by computer-generated perceptual information. VR and AR differ in that VR replaces the real world with an artificial one, while AR does not cut the user off from reality. A person engaging with a VR headset, for example, only visualizes what is happening inside the headset, while the AR headset enhances aspects of the individual’s physical environment [2,3]. In MR, virtual objects and information are not just superimposed onto the physical environment but interact with and respond to it. This interaction creates a more dynamic and integrated experience, allowing digital and physical elements to coexist and interact in real time. 

Surgical simulation and skill training using enhanced reality devices offer an opportunity to teach, practice, and assess technical proficiency without using actual patients. Such tools can reduce the learning curve for developing neurosurgeons, improve conceptual understanding of complex anatomy, and enhance visuospatial skills [4]. However, the use of these technologies is dependent on the adoption of specialized equipment and equipment in order to leverage such applications [5]. General applications of VR, AR, and MR include improvising diagnostics, enabling surgical navigation, and training the residents for surgical execution [4].

Recent technological advances, including 3D microscopy and endoscopy, robotics, advanced neuroimaging, and artificial intelligence, have continued to advance the surgeon–computer relationship. The aim of this study was to review the current applications of VR, AR, and MR for surgical training, surgical execution, and patient education, with a primary focus on spine surgery. The purpose of this study is to provide an outline of the current state of these technologies so that future optimization and implementation may be proposed.

## 2. Materials and Methods

### 2.1. Data Sources and Search Strategy

We prespecified the methods used in this systematic review and presented them in accordance with PRISMA (Preferred Reporting Items for Systematic Reviews and Meta-Analyses) guidelines (Supplementary File S1). The systematic review protocol was registered with the Open Science Foundation (https://doi.org/10.17605/OSF.IO/72YJE, accessed on 30 May 2021). On 23 January 2023, a literature search was performed using PubMed and Scopus (Appendix A). Only articles written in English were reviewed. All human, cadaveric, and phantom studies that utilized VR, AR, or MR for spine surgery with applications for surgical training, education, assessment, and patient education were included. Articles were excluded if they were not related to VR, AR, MR, and spine surgery, or if they were technical reports of devices without a tested application in surgical training, surgical execution, or patient education.

### 2.2. Data Extraction and Quality Assessments

Data were extracted according to whether the study was conducted on artificial models (phantoms/sawbones), human cadavers, or patients. To evaluate the quality of the studies, we employed the validated Medical Education Research Study Quality Instrument (MERSQI) for studies assessing the ability of an intervention to increase the success rates of surgical trainees. For cadaveric studies, we employed the Quality Appraisal for Cadaveric Studies (QUACS) scale. For clinical studies, we utilized the Joanna Briggs Institute (JBI) critical appraisal tools that permit the evaluation of the quality of studies for case reports, case series, observational studies, and randomized clinical trials [6]. Two reviewers performed quality assessments [7,8]. Articles were grouped according to the application to be discussed as follows: surgical training, surgical execution, and patient education. 

## 3. Results

### 3.1. Selected Articles

**Figure 1:**
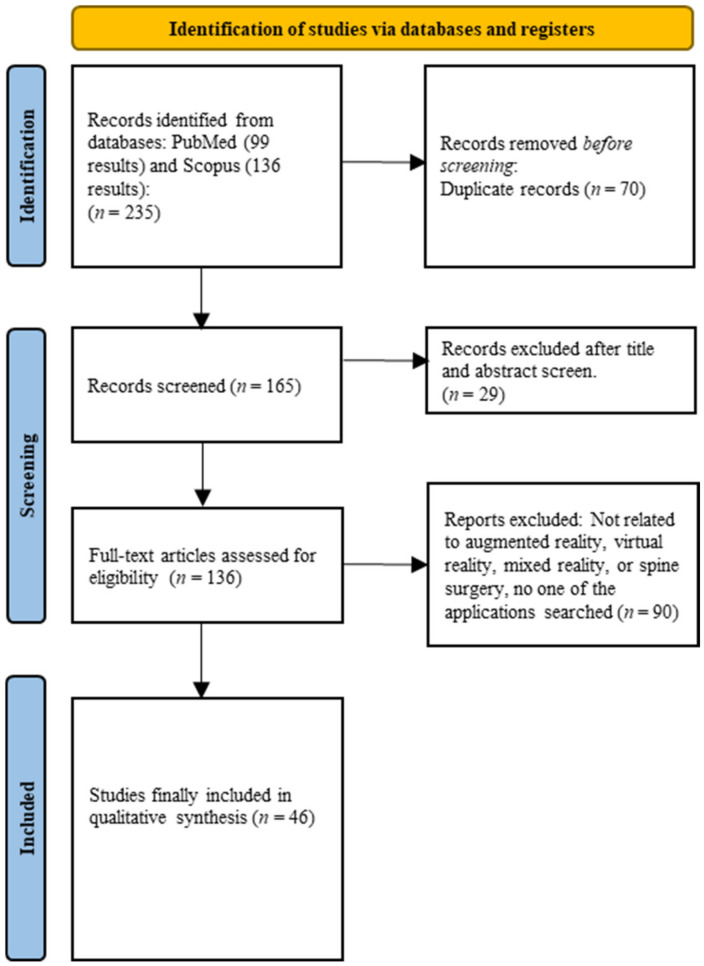
PRISMA flow diagram demonstrating the study identification and full-text article selection process.

The initial literature search yielded 228 citations. After deleting duplicate citations, titles, and abstracts, 136 unique articles were screened. Next, the full texts of articles were evaluated, of which 46 articles fulfilled our criteria and were included in this review. A total of 14 studies discussed VR applications, 27 discussed AR applications, and 5 discussed MR applications. Figure 1 highlights the study selection process. 

### 3.2. Characteristics of the Included Studies, Study Settings, and Data Synthesis

Table 1 reports the main characteristics of all the 46 included studies (grouped by application). We ultimately included 9 studies using phantoms (6 case series and 3 parallel randomized trials), 9 studies using cadavers (1 case study, 6 case series, and 1 parallel randomized trial), and 28 studies in humans (4 case studies, 19 case series, 1 case–control study, and 4 parallel control trials). A total of 9 out of the 46 studies involved VR, AR, or MR used for educational purposes. The hardware through which AR, VR, MR were delivered included monitors, glasses, headsets, and microscopes. The most simulated surgical procedure was percutaneous pedicle screw placement; in one simulation, critical care scenarios were simulated as opposed to a specific surgical procedure.

### 3.3. Study Quality and Level of Evidence

For nine articles regarding educational/instructional interventions, we used the MERSQI checklist to demonstrate considerable variation among studies. The average total MERSQI score of the eight studies was M = 11.6 out of 18 (SD 3.2), thereby indicating a moderate level of quality for educational initiatives. The average QUACS score for the nine cadaveric studies was 11.11 out of 13 (SC 0.99), thereby indicating high-quality cadaveric studies. The JBI critical appraisal for clinical observational studies (case studies, case series, case–control studies) ranged from moderate to low quality, and for randomized controlled trials it ranged from moderate to low quality. No pooled statistical analyses were performed given the heterogeneity of the study designs, interventions, and reported outcomes of the studies. Scores per study are found in Supplementary File S2.

## 4. Discussion

### 4.1. VR Applications for Surgical Simulation and Execution in Spine Surgery

#### 4.1.1. Teaching

We compiled eight studies that evaluated the performance of VR/AR models for teaching procedures to neurosurgical trainees and surgeons [9,10,11,12,13,14,17,18]. The devices, technology used, and procedures differed across all the studies. The simulated procedures included percutaneous spinal needle placement [9], percutaneous lumbar pedicle screw placement [10,13], percutaneous transforaminal endoscopic discectomies [17], and lateral lumbar access to the spine [17].

Three randomized trials were identified to evaluate the efficacy of AR/VR for training purposes. Gasco et al. explored the usefulness of the Immersive Touch VR Simulator (ImmersiveTouch^®^, ImmerseTouch Inc., Chicago, IL, USA) against traditional visual and verbal instructions for pedicle screw placement [10]. This study included 26 senior medical student participants randomized into two groups, which were with and without simulation. They were then asked to place pedicle screws in lumbar sawbones models. The VR-trained group outperformed the non-trained group in the average number of errors per screw across 52 analyzed screws.

Another study by Xin et al. studied whether the pedicle screw placement (PSP) skills of young surgeons could be improved effectively after receiving immersive VR surgical simulator (IVRSS) training [13]. The success rate and accuracy rate of screw placement in the VR group and the non-VR group were 82.9% and 69.6% vs. 74.2% and 55.4%, respectively, showing statistically significant differences between the two groups.

Thirdly, Hou et al. utilized a randomized control cadaveric study to investigate the effectiveness of a virtual surgical training system (VSTS) on resident performance for cervical pedicle screw instrumentation [18]. Ten residents were randomly assigned to two groups as follows: one group received VR-simulated surgical training and the control group was given an introductory teaching session before the procedure. The rates of the properly placed screws were 90% for the AR-trained group and 37.5% for the control group, further demonstrating VR to be an effective instructional tool.

As medical education becomes more complex, cost-effective, and straightforward, simulators that facilitate the development of trainee skills and knowledge outside the operating room are increasingly necessary [11]. For example, minimally invasive spine surgery (MISS) involves complex motor skills in regions of variable anatomy. Thus, VR may aid in training, skill retention, preoperative planning, and intraoperative execution. Randomized controlled studies have shown VR simulators to routinely outperform traditional methods of training for MISS procedures, including pedicle screw insertion [10,13,18] and percutaneous discectomies [17], for both multistep procedural components and entire surgeries [12,55,56]. Because VR-based systems can accurately simulate surgical scenarios, patients are not harmed when mistakes occur. Residents can also repeatedly practice surgeries, which offers a unique training advantage [57,58]. The main current limitation of VR educational platforms is a lack of haptic feedback, which limits sensory-dependent learning such as feeling tactile resistance during surgery [12].

#### 4.1.2. Surgical Planning

Because some surgeons plan their surgeries using 3D printed bone models, having VR reconstructions that could simulate bony anatomy in the same way might serve as an efficient alternative model. As a proof of concept, De Salvatore et al. applied a Google Cardboard-based VR system for the patient-specific preoperative planning of adolescent scoliosis surgery and demonstrated significant decreases in the operative time and bleeding [50]. A case series by Croci et al. also highlighted the ability of SpectoVR, a 3D visualization software, to apply patient-specific, virtual 3D holograms for surgical preparation purposes [14]. VR perspectives were shown to significantly influence recommendations of surgical therapies and techniques in spinal fusion, so a natural evolution of these technologies may include implementing the ability to focus on soft tissue anatomy for consultation purposes [54]. For example, the surgical planner from surgical theater processes information from patient records to create patient-specific VR reconstructions for a neurosurgeon to comprehensively visualize intracranial lesions, brain parenchyma, and intramedullary spinal cord neoplasms [52,59]. There is potential for further application of this platform in microsurgical spine applications to microdiscectomies, intradural tumor resections, and vascular lesion repair [52]. The results of a preliminary evaluation of this developed prototype in the context of surgical planning should include the consideration of the benefits and limitations that could support future design efforts in spine surgery simulations [60].

#### 4.1.3. Assessment Tools

VR-based assessment tools using simulations can play an essential role in the emergency care training of first responders and clinicians in cranial and spinal surgery. NeuroVR™ (CAE Healthcare, Sarasota, FL, USA) is a VR-based neurological simulator that allows for the bimanual manipulation of cranial models and the practice of standardized tasks in a stereoscopic view, thereby providing specific metrics and quantitative measurements [61] and simulating surgical procedure and measuring performance through standardized scores. NeuroVR™ (CAE Healthcare, Sarasota, FL, USA) may constitute a useful and powerful tool for acquiring, improving, and assessing neurosurgical competencies. It can simulate multiple fundamental neurosurgical skills including microdissections, tumor aspiration, and hemostasis [2] for high-stake assessments [1]. Another simulator by serious games platforms (SGPs) utilizes virtual standardized patients (VSPs) to develop situational awareness for medical trainees and is increasingly used in healthcare training and assessment [1,62]. A study performed by Stefan et al. further displayed accurate skill discrimination when applying computer-assisted, simulated reality for surgical assessment; they successfully associated good, simulated scores with established standardized metrics in vertebroplasty [15]. The future development of a virtual and physical simulator for spine surgery to provide practice for pedicle screw placement and lumbar stenosis decompression surgery remains a promising alternative to surgical rehearsal for assessing technical feasibility and skill [63]. One such example is that using VRSpineSim, a 3D stereoscopic VR spine simulation with unique educational features and simplified interactions designed to support surgeons with a convenient environment to learn and rehearse pedicle screw insertion [60].

#### 4.1.4. Patient Education

We did not find any specific examples of VR platforms to conduct patient education specifically for spinal surgeries. However, a study evaluated the influence of a personal digital assistant (PDA) on patient education in a clinical setting. The outcome measures were determined by monitoring distinct factors such as those of the participant’s knowledge of the disease and medications, self-reported adherence, and the practicality of the intervention. The results showed that technology-assisted education through PDA was a convenient and powerful method of delivering health messages to patients [64]. A natural progression of this technology may be through the application of VR to improve patient communication by providing education in a comprehensively accessible and visually coordinated manner. For example, a physician could use the 3D models generated for surgical planning to explain procedures for patients and family in a visually palatable manner.

VR-based therapy—from home-based non-immersive interventions to high-technology immersive interventions requiring sensitive or technically advanced software—may be applied to achieve a positive effect perioperatively and in rehabilitation [47,65]. A randomized control trial by Bekelis et al. showed increased patient satisfaction perioperatively when exposed to preoperative VR [47]. Meanwhile, Sengupta et al. explored VR’s potential in the context of balance training for patients with spinal cord injury; this technology could potentially be applied for patients undergoing post-surgical rehabilitation as well [65]. Another post-surgical application of a VR-based 3D slicer software was that which demonstrated by Alsofy et al. of clinically assessing postoperative complications of a stand-alone cage insertion for patients suffering from kyphosis. They reported an improvement in the ease and accuracy of radiographic evaluations when VR was implemented [38]. Similarly, Su et al. developed a VR-based clinical assessment tool (myelopathy-hand functional evaluation system) to evaluate hand dysfunction in patients with cervical myelopathy [46]. Hence, VR can be effectively used to improve surgical outcomes, postoperative assessment, and rehabilitation.

### 4.2. AR Applications in Surgical Simulation and Execution in Spine Surgery

#### 4.2.1. Teaching

To date, AR has primarily been used to superimpose images over a live view of the human anatomy. AR was first introduced to surgery with the C-arm/O-arm-based computerized spinal navigation for pedicle screw placement. Because this system projected super-imposed images on a workstation monitor, ergonomically challenging requirements manifested as surgeons needed to look at both the operative field and AR display. The 12 studies that we found regarding AR and spine surgery all sought to find an efficient way to overlay CT or MR images over the operative field to address this limitation. In a study published in the Journal of *Academic Radiology*, medical students were able to learn anatomy significantly better when using AR systems [66]. The Insight Heart system from Magic Leap (Magic Leap, Florida) provides a detailed, unorthodox method to explore human anatomy. The system scans the physical surroundings, and a 3D body is projected, which students may study in detail [67]. Another AR system, the Anatomy X (Medivis, New York, NY, USA) component of Medivis HoloLens by Microsoft, is an AR anatomy lab learning platform that provides high-definition models, multi-user sharing, and comprehensive anatomic information [68]. Within spine surgery, the IEEE (Institute of Electrical and Electronics Engineers) reported development of an AR-based system that enables training for minimally invasive spine surgery by providing the trainee with real force feedback using a real training instrument, 3D physical spine models, and two infrared cameras. It also has a standardized scoring system that can assess the trainee’s performance against that of an expert, which allows for continuous skill refinement [69].

#### 4.2.2. Surgical Execution

While VR platforms are better for immersive surgical planning and rehearsal environments, AR platforms are generally better suited to surgical execution. AR-based navigation is versatile and offers an intuitive operating experience. It mitigates the need to mentally relate neuro-navigational data to the patient’s anatomy, permitting surgeons to continuously maintain their attention on the surgical field [16,70,71]. Augmedics’ xVision (Augmedics, Chicago), at the time of this writing, is the only FDA-approved AR platform to guide thoracolumbar pedicle screw placement. This platform utilizes a head-mounted passive infrared tracking camera to provide stereotactic navigation through a direct retinal display that overlays navigational data directly over the surgical field [72]. This AR system requires no additional equipment around the operating table, allowing for direct and unobstructed line of sight, which has proven beneficial for pedicle screw insertions [19,73]. The effectiveness of AR-mediated pedicle screw placements was topographically reviewed with follow-up CT scans [45,53].

In a cadaveric proof-of-concept study by Urakov et al., thoracic pedicle instrumentation was performed using either fluoroscopy or AR equipment and software. While fluoroscopic methods yielded greater pedicle screw insertion accuracy, the study showed AR screws to maintain correct directionality and parallel orientation relative to the desired trajectories [19]. Molina et al. further explored the use of AR navigation with Augmedics’ xVision (Augmedics, Chicago, IL, USA) [20,21]. One of these studies assessed the comparative accuracy of AR-assisted pedicle screw insertion compared with conventional pedicle screw insertions in cadavers. Screw insertion accuracy was assessed from postoperative CT scans. Overall screw placement accuracy achieved using AR was 96.7% based on HGS (Heary–Gertzbein scale) and 94.6% based on GS (Gertzbein scale) grading scales [20]. The same researchers evaluated the accuracy and precision of AR-HMD on percutaneous AR-assisted pedicle screw insertion. The overall clinical accuracy achieved was 99.1% using the AR system [21]. Similar cadaveric AR pedicle screw studies by Peh et al., Elmi Terander et al., Siemionow et al., and Chang et al. reported the freedom to improvise on surgical navigation, facilitating s smooth surgical workflow and improved accuracy rates when compared with fluoroscopy [22,24,25,26]. Meanwhile, Burström et al. explored the use of adhesive skin markers placed surrounding the surgical field for AR-based pedicle screw installation. They only found negligible differences in the technical accuracy between the vertebral levels. This finding suggests that the current practice of relying on dynamic reference frames that are obstructive and require continuous adjustment between vertebra may be improved through the application of augmented reality surgical navigation. In this sense, AR may further improve ergonomic and visual-field benefits [48].

Several in-human studies of AR-based navigation have also been reported. Molina et al. reported utilizing xVision to conduct an L4-S1 decompression, pedicle screw insertion, and rod fixation on a 78-year-old woman with degenerative spine disease. The study reported a 100% Gertzbein score pedicle screw insertional accuracy without any associated complications [34]. Yahanda et al. similarly reported a 100% accuracy rate according to the Gertzbein–Robbins grade for the insertion of 63 percutaneous pedicle screws in nine patients guided by ARHMD technology [36]. Molina et al. also reported the utilization of xVision on a 69-year-old male for guidance in a unique osteotomy execution to achieve the en bloc wide marginal resection of an L1 chordoma through a posterior-only approach [35]. In this report, AR-HMD allowed the surgeon performing the osteotomy to simultaneously visualize the navigational guidance provided by the contralateral surgeon’s tracked pointer and the progress of an ultrasonic bone saw aligned parallel to the tracked instruments. This permitted the execution of disc and bone cuts that avoided the tumor capsule while minimizing exposure and collateral tissue damage; the AR-HMD enabled a less invasive successful en bloc resection of this lesion [35]. A retrospective review by Liu et al. also reported the clinical accuracy of AR-assisted pedicle screw placements in 28 patients within the thoracic, lumbar, and/or sacral spine to be 98.5%, 97.8%, and 98.0% on the Gertzbein–Robbins scale, respectively, further supporting the patency of the method [37]. Gibby et al. similarly found that 18 AR-mediated procedures in 10 patients had statistically indifferent error rates from 32 in the control, non-AR phantom models [30].

Further AR support has also been applied to supplement intraoperative CT/fluoroscopy-based navigation for unorthodox approaches and minimally invasive procedures that have resulted in decreased blood loss, operative time, and postoperative pain. This was accomplished by visualizing tumor outlines, pedicle screws, herniated discs, and surrounding structures for lateral and transforaminal spinal approaches in living patients [43,49]. One case was reported to have even applied AR usage for endoscopic transforaminal interbody fusion [51].

These current navigation systems are implant-agonistic, which allows for the use of cost-effective implants that are best suited for the patient and the surgeon, potentially leading to reduced costs. They can also be easily adapted for either open or minimally invasive applications using the same registration approach [73]. Furthermore, the Augmedics xVision is priced at $150,000, which is significantly less costly than currently available manual and robotic systems that range from USD 400,000 to 1,000,000 [74,75]. An even more affordable “do-it-yourself” version of the AR HUD product with the capability of overlaying 3D or fluoroscopy images onto a surgeon’s field of view may also be fashioned for under $1000 from commercially available products [40].

As AR becomes more widely available in spine surgery, AR-trained physicians must make efforts to avoid becoming over-reliant on these technologies. Depending on an institution’s technological availability during emergent situations, procedures that may otherwise be performed with AR may require unassisted execution. Research surrounding AR over-reliance in spine surgery is limited, but a study on spinal navigation by Kaliya-Perumal et al. found that trainees were overall better trained in pedicle screw placement with neuronavigation, yet some residents were unable to correctly identify entry points without assistance [76].

#### 4.2.3. Microscope-Based AR

AR-based microscopy is another potentially powerful tool for spinal procedures because it improves anatomical orientation in the surgical field, provides radiation-free patient registration, and provides comfort for surgeons without interrupting the surgical workflow [28]. A study by Carl et al. utilized the heads-up displays of certain operating microscopes (Pentero/Pentero900, Zeiss, Oberkochen, Germany) for AR support. The system integrated preoperative CT, MRI, and PET as well as intraoperative imaging to visualize 3D objects in a semi-transparent or solid mode superimposed over a microscope video. They used the generated images to assist 10 different spinal surgeries including laminectomy, laminoplasty, posterior fixation, and corpectomy. This workflow resulted in high navigation accuracy with a mean registration error of about 1 mm [27]. In a posterior study, this group performed a series of degenerative spine surgeries. This microscope-based AR system allowed for the superior visualization of and orientation around the target structures. Moreover, this digital content was not only displayed on top of the real-world image but was also interactive alongside the real-world image, providing a merged reality [28]. After continued use, the authors were able to use this system to complete 42 different spine procedures (12 intra- and 8 extradural tumors, 7 other intradural lesions, 11 degenerative cases, 2 infections, and 2 deformities) [29]. In all cases, landmark checks demonstrated high registration accuracy and exhibited the benefit of this microscope-based AR system, especially in challenging anatomical situations. In a different study, Farshad et al. also reported reduced leg pain and signs of radiculopathy resolution in the first human case where direct holographic navigation was used for pedicle screw placement, further supporting the use of AR in spine surgery [39].

Felix et al. expanded the field of AR microscopy to minimally invasive spinal surgeries in a cadaveric concept study. They demonstrated 96% accuracy in screw placements using VisAR microscopy [23]. A similar study by Buch et al. used intraoperative holographic models to register landmarks during spinal fusion surgery as a proof of concept that must also be further explored [44]. Another study used intraoperative cone beam CT scan combined with Allura ARSN in a controlled study for spinal fixation surgeries aiming to reduce the need for a postoperative CT scan as well as the accurate placement of pedicle screws [77]. Thus, AR-based technology has been demonstrated to pave the road for novel surgical holographic navigation. Although AR clinical adoption in spine surgery has increased since the conduction of the first human case, most publications in the literature include non-controlled and non-randomized studies, so future studies on the impact of clinical outcomes, such as patient mortality, morbidity, and complications, are required [77].

#### 4.2.4. Patient Education

We did not find any specific examples of AR platforms employed in neurosurgical patient education encounters. However, Patient AR is a platform used for patient education in the setting of orthopedic joint procedures. These systems are designed in a manner where a doctor and patient can both visually review the surgical plan and understand the outcomes and risks of the procedure through a non-occlusive mixed reality setting [78]. A review of the capabilities of the platform demonstrates its feasibility and potential adoption for spine pathology provider–patient education encounters.

### 4.3. MR Applications for Surgical Simulation and Execution in Spine Surgery

#### 4.3.1. Surgical Execution

Novel innovations using mixed reality are also being applied for spinal surgery. Promising results suggest that the application of MR for surgery yield cost and safety benefits. In a publication by Aoyama et al., a case series of two patients undergoing spinal decompression has shown that the preoperative confirmation of the decompression area using a MR-assisted head-mounted device could reduce the risk, time, and cost of the procedure [41]. Additionally, Gu et al. demonstrated a reduction in the risk, operative time, radiation exposure, and bleeding in patients who had undergone lumbar pedicle screw placement with HoloLens technology used with MR compared with patients who had undergone traditional C-arm fluoroscopy methods [42]. Buch et al. further established the applicability of MR in spinal fusion by developing a method of creating and iteratively optimizing holographic model constructs from patient landmarks in a real-time, intraoperative setting [44]. While such initial results illustrate the potential benefits associated with MR technological applications for spinal surgeries, further research must be conducted on a larger scale to develop a solid pipeline for its implementation.

#### 4.3.2. Teaching and Patient Education

We did not find any specific examples of MR platforms to conduct patient education and neurosurgical simulations specifically for spinal surgeries.

### 4.4. Limitations

The first limitation refers to the focus on English-language articles in our systematic review. We may have missed additional studies published in technical journals as our focus was on already tested clinical or training applications. All the identified studies reported positive findings; therefore, results must be interpreted carefully. The presence of nonrandomized designs, the lack of control groups and long-term follow-ups, and the poor reporting of the study methods and outcomes constitute the main shortcoming of the included studies, which was reflected in the quality assessment of the reviewed articles. Regarding the extracted data of the study, there is a general lack of literature regarding the actual efficacy and applicability of many of the technological advances in spine surgery. However, several studies demonstrate the potential for these tools to be applied for practice in spine surgery. Future research on these technologies should aim to further lower the barriers to the adoption of navigation technologies, thereby increasing access to high-quality educational platforms to trainees, providing reproducible, fair platforms to assess trainee skills, and providing immersive, intuitive environments to educate patients and their advocates.

## 5. Conclusions

The combination of VR with dynamic, 3D stereoscopic visualization and haptic feedback technologies makes realistic procedural simulations possible. Most neurosurgical procedures can be conceptualized and segmented into critical task components, which can be simulated independently or in conjunction with other modules to recreate the experience of a complex neurosurgical procedure in a low-stake learning environment [79]. VR can thus teach surgeons new procedures and determine their level of competence before they operate on patients. Surgical simulation will likely play a vital role in the future, and quantitative measurements of competence through these platforms will likely be used to assess and shorten spine surgical training [80]. VR platforms also provide powerful platforms for patient education. There are currently various technologies that use virtual reality to enhance patient education through immersive devices. They have the potential to provide patients with self-driven, in-depth knowledge about their medical condition and reduce the healthcare system’s workload. They also have the potential of enhancing patient–doctor communication and patient understanding, which may lead to higher patient satisfaction.

AR platforms have greater potential as an adjunct to live surgeries as they allow the surgeon to better understand 3D patient anatomy while operating [70]. AR head-mounted displays employed in spine surgery have several potential advantages, including the improvement of the line-of-sight limitations present in conventional manual and robotic computer navigations and the reduction of the user learning curve because of the intuitive overlaying of navigation data directly onto the surgical field. Beyond decreasing the learning curve to adopt stereotactic spine navigation, the systems also provide a significant cost advantage in comparison with traditional manual and robotic computer navigation systems [81]. As the demand for stereotactic navigation in spine surgery increases, it becomes increasingly important to assess and research technologies that maximize accuracy, precision, and efficiency but that also minimize costs. AR platforms are poised to serve these promising goals in spine surgery [81].

The utilization of mixed reality (MR) in spinal surgery presents promising advancements with evident benefits in terms of cost and safety. The innovative application of MR, as demonstrated in the cited studies, highlights its potential to reduce risks, operative time, radiation exposure, and overall procedural costs. Notably, the use of MR-assisted head-mounted devices has proven effective in enhancing pre-operative planning and intraoperative navigation for spinal procedures. While initial case series and studies highlight the positive impact of MR in spinal surgeries; at its current stage and compared with AR, MR may not add a different value to spinal surgery. Establishing a robust data pipeline that ensures accuracy is a necessary step before any other MR applications can be developed for spinal surgery.

## Figures and Tables

**Table 1:** Referenced Literature Overview and Quality Assessment.

Study	Citation Number	Rendered Model/Cadaver/Human	Study Aim	Research Subjects	Study Design	Device	Tech	Activity Simulated	Outcome Measures	Main Findings	Quality Measurement Tool/Outcome
Luciano et al., 2013	[9]	Phantom	To experimentally determine learning effectiveness in terms of entry point/target point accuracy of percutaneous spinal needle placement.	63 participants	Case series	Immersive touch	VR	Percutaneous spinal needle placement.	Performance error; fluoroscopy times.	Usage of high-performance AR and haptic technology simulation resulted in an initial failure rate of 7.93%; average error, fluoroscopy exposure, and individual performance scores improved from the first to the second attempt	MERSQI/11.5
Gasco et al., 2014	[10]	Phantom	To explore the usefulness of virtual simulation training for learning to place pedicle screws in the lumbar spine with 26 senior medical students.	26 participants	RCT	Immersive touch simulator	VR	Pedicle screw placement.	Errors per screw.	Simulation train.ing resulted in a 53.7% error reduction, with an average number of errors per screw of 0.96 vs. 2.08 in the non-simulation group. The simulation group performed better than the non-simulation group in all measured variables.	MERSQI/15.5
Lorias-Espinoza et al., 2016	[11]	Phantom	To propose a designed low-cost spinal surgery simulator (Spine MovDigSys 01).	12 participants	Case series	Spine MovDIGSys01	VR	Needle insertion.	Assessment matrix.	The simulator system was tested based on operator metrics to demonstrate educational value for transforaminal endoscopic discectomy.	MERSQI/9
Luca et al., 2020	[12]	Phantom	To demonstrate the potentiality of VR phantom simulation training through its application for spine surgery.	10 participants	Case series	Oculus VR	VR	Lateral lumbar access to the spine.	Procedural time and number of errors.	Residents and junior orthopedic surgeons demonstrated improvements after a second attempt in separate training settings. Major errors decreased from 5.2 to 1.8 in the preoperative setting and from 4 to 1.4 in the surgical simulation setting.	MERSQI/13.5
Xin et al., 2020	[13]	Phantom	To verify whether the pedicle screw placement (PSP) skills of young surgeons receiving immersive virtual reality surgical simulator (IVRSS) training could be improved effectively and whether the IVRSS-PSP training mode could produce real clinical value for clinical surgery.	24 participants	RCT	Immersive virtual reality surgery simulator (IVRSS)	VR	Pedicle screw placement.	Success rate and accuracy rate of screw placement.	The success rate of bare-handed nailing was about 69.2% for surgeons before IVRSS-PSP training vs. 82.9% after IVRSS-PSP training.	MERSQI/15.5
Croci et al., 2020	[14]	Phantom	To report the preoperative use a of novel platform (SpectoVR) that presents DICOM data as virtual 3D holograms.	8 patients	Case series	SpectoVR	VR	Surgical planning to correct cervical deformities, stenosis, and tumor resection.	Awareness about case complexity.	The surgeons felt that SpectoVR substantially improved their situation awareness about each case. Integrating SpectoVR into daily presurgical planning is feasible.	MERSQI/8
Stefan et al., 2023	[15]	Phantom	To assess the application of universal framework for computer assisted simulated workplace-based assessment in surgery.	11 Surgeons	Case series	Simulated workplace-based assessment	VR	Various kinds of surgeries.	Performance evaluation compared with job experience.	Simulated workplace-based assessment scores accurately quantified surgeons’ experience, observational assessment scores, and overall pass/fail rates.	MERSQI/8
Babichenko et al., 2023	[16]	Phantom	To determine whether the mere presence of a Microsoft HoloLens AR headset would prohibitively affect extraneous cognitive load (ECL) during a simulated surgical procedure.	22 Surgeons	Case series	Microsoft HoloLens	MR	Screw placements.	Median drilling times and screw placement times.	No significant differences presented in cognitive load when trials with the HoloLens 1 in comparison to the trials without the HoloLens 1. Cognitive load was measured with the SURG-TLX questionnaire and surgical performance metrics.	MERSQI/8
Yu et al., 2019	[17]	Phantom	To explore the effect of preoperative planning using MR on training throguh percutaneous transforaminal endoscopic discectomy.	60 participants	RCT	3D Slicer platform	MR	Percutaneous discectomy.	Acceptance rates of MR technology. Puncture times, finish times, and fluoroscopy times.	Times of puncture and fluoroscopy and finish times (minutes) were significantly decreased by preoperative planning based on MR compared with traditional methods.	MERSQI/15.5
Hou et al., 2018	[18]	Cadaveric	To investigate the effectiveness of a virtual surgical training system (VSTS) on cervical pedicle screw instrumentation for residents.	10 cadavers	RCT	Virtual surgical training system	VR	Cervical pedicle screw placement.	Screw penetration rates and average screw penetration distance.	The simulation group and control group had a significantl difference in screw penetration rates of 10% and 62.5%, respectively; they also had a significant difference in average screw penetration distances of 1.12 mm and 2.08 mm, respectively.	QUACS/10
Urakov et al., 2019	[19]	Cadaveric	To provide a demonstration of a workflow in the application of AR in the setting of spine surgery.	1 cadaver	Case study	HoloLens AR glasses	AR	Percutaneous pedicle screw placement.	Breach and accuracy	Seven major breaches were found with AR-mediated screw insertion; no major breaches were found with fluoroscopy-assisted pedicle instrumentation.	QUACS/9
Molina et al., 2019	[20]	Cadaveric	To assess the comparative accuracy of AR-assisted pedicle screw insertion in comparison with conventional pedicle screw insertion methods.	5 cadavers	Case series	X Vision, AR-HMD	AR	Open pedicle screw placements.	Gertzbein–Robbins scale, Heary–Gertzbein scale, and accuracy rates.	AR screw placement accuracy was 96.7% based on HGS scores and 99.6% based on GS; conventional navigation scores were non-inferior for both HGS and GS.	QUACS/12
Molina et al., 2020	[21]	Cadaveric	To assess the accuracy and precision of percutaneous ARMSS pedicle implant insertion.	5 cadavers	Case series	X Vision, AR-HMD	AR	Percutaneous pedicle screw placement.	Mean screw tip linear deviation and mean angular error.	AR-mediated lumbosacral and thoracic pedicle implant accuracy were 100 and 98.2%, respectively. Overall implant accuracy was 99.1%.	QUACS/12
Peh et al., 2019	[22]	Cadaveric	To determine the accuracy and feasibility of ARSN for minimally invasive thoracic and lumbar pedicle screw placement.	4 cadavers	Case series	ARSN	AR	Open pedicle screw placements.	Gertzbein—Robbins scale.	No significant difference was found between thoracic and lumbar pedicle screw placements with ARSN vs. fluoroscopy, with overall accuracies of 94% vs. 88%.	QUACS/12
Felix et al., 2022	[23]	Cadaveric	To determine the accuracy of pedicle screw placement using VisAR for open spine and MIS spine procedures.	7 cadavers	Case series	VisAR	AR	Open and percutaneous pedicle screw placement.	Gertzbein—Robbins Scale.	A total of 24 pedicle screws were inserted using VisAR with a combined accuracy of 96%.	QUACS11
Elmi Terander et al., 2018	[24]	Cadaveric	To assess the feasibility and accuracy of minimally invasive thoracolumbar pedicle screw placement using ARSN.	3 cadavers	Case series	Philips augmented reality surgical navigation system	AR	Percutaneous pedicle screw placements.	Distance between the tip of the actual needle position, angles between the needle, and the desired path	Thoracolumbar MIS screw placement directed by AR with intraoperative 3D imaging in a hybrid OR was 89% accurate.	QUACS/11
Siemionow et al., 2020	[25]	Cadaveric	To develop a technique and assess the accuracy and feasibility of lumbar vertebrae pedicle instrumentation on using ARSN.	4 cadavers	Case series	ARAI-assisted surgical navigation	AR	Pedicle screw placement.	Gertzbein score, Ravi score, Zdichavsky score.	ARAI-assisted surgical navigation correctly placed probes for pedicle screw insertion compared with 3D generated images from intraoperative scans.	QUACS/11
Chang et al., 2022	[26]	Cadaveric	To validate the feasibility and accuracy of augmented reality (AR)- assisted percutaneous pedicle screw instrumentation.	1 cadaver	Case study	HMD AR glasses	AR	Feasibility and accuracy of pedicle screw placement.	Gertzbein–Robbins Scale.	AR-assisted percutaneous pedicle screw instrumentation was 87.5% accurate through the GR scale.	QUACS/12
Carl. et al., 2019	[27]	Human	To implement a workflow that allowed for the establishment of a microscope-based augmented reality (AR) environment to support spine surgery.	10 patients	Case series	A microscope-based AR	AR	Laminectomy, laminoplasty, losterior fixation, corpectomy.	Registration accuracy and surgery success.	Automatic microscope-based AR patient registration resulted in high navigation accuracy with a mean registration error of about 1 mm and added only about 5 min to the surgical procedure.	JBI Case-series/8
Carl et al., 2019	[28]	Human	To establish microscope-based AR support (head-up display—HUD) for degenerative spine surgery.	10 patients	Case series	Microscope-based AR support	AR	Anterior, lateral, posterior median, and posterior paramedian approaches for degenerative spine surgery.	Registration/visualization accuracy, accuracy, time, or radiation.	Microscope-based AR support had a target registration error of 1.11 mm, with 1/3 of the standard radiation dose of a diagnostic spine scan.	JBI Case-series/8
Carl et al., 2020	[29]	Human	To investigate how microscope-based augmented reality (AR) support can be utilized in various types of spine surgery.	42 patients	Case series	Microscope-based AR support	AR	Anterior, lateral, posterior median, and posterior paramedian approaches for degenerative spine surgery as well as intradural and extradural tumor resections.	Feasibility, registration accuracy, and radiation exposure.	AR smoothly supported various kinds of spine procedures and facilitated anatomical orientation in the surgical field. On average, 7.1 objects were reliably displayed, visualizing target and risk structures.	JBI Case-series/8
Gibby et al., 2020	[30]	Human	To demonstrate the usefulness of the HoloLens-mounted AR display to guide and perform percutaneous spine procedures.	10 patients	Case series	HoloLens HMD	AR	Percutaneous discectomy, sacroplasty, lumbar facet joint injection, and lumbar puncture.	Need of realignment and error of needle to target	The success rate of performing a procedure not requiring any realignment was 82.35%. The mean error of needle to target was 1.73 mm.	JBI Case-series/5
Edström et al., 2020	[31]	Human	To present a workflow for an ARSN system installed in a hybrid operating room.	20 patients	Case series	A C-arm flat panel detector modified with 4 optical video cameras	AR	Pedicle screw placement.	Total procedure time.	Intraoperative imaging and preparation for surgical navigation totaled 8% of the surgical time. These results suggest that ARSN may be used to minimize radiation exposure.	JBI Case-series/3
Hu et al., 2020	[32]	Human	To demonstrate that the augmented reality computer-assisted spine surgery (ARCASS) system is clinically feasible for percutaneous vertebroplasty.	18 patients	Case control	ARCASS system (combination of a C-arm, an industrial camera, and a high-quality projector)	AR	Percutaneous vertebroplasty.	Operative time, frequency of fluoroscopy, accuracy for lateral and anteroposterior view. Oswestry Disability Index.	Compared with the control, ARCASS had fewer uses of fluoroscopy (6 vs. 18 times), shorter operative times (78 vs. 205 s), and better entry point identification (with 81.8% vs. 30.0% deemed good for lateral views and 72.7% vs. 20.0% for anteroposterior views ).	JBI Case-control/7
Auloge et al., 2020	[33]	Human	To evaluate technical feasibility, accuracy, safety and patient radiation exposure by a novel navigational tool during percutaneous vertebroplasty.	20 patients	RCT	Four small video cameras integrated within the frame of the x-ray detector of a C-arm	AR	Percutaneous vertebroplasty.	Technical feasibility, accuracy, procedural safety, time for trocar placement, and patient radiation exposure.	AI software successfully identified landmarks and generated a safe/accurate trajectory consistently. Accuracy of trocar placement was similar between fluoroscopy and AI methods. Patient radiation dose during the targeting phase was 50% lower with AR/AI guidance compared with fluoroscopy alone.	JBI RCT/10
Molina et al., 2020	[34]	Human	To report a technical note, accuracy, and precision analysis of the first in-human deployment of this technology.	1 patient	Case study	X Vision, AR-HMD	AR	Pedicle screw placement.	Mean linear deviation and mean angular deviation.	Clinical accuracy of screw placement was 100% through a GS grading scale.	JBI Case-study/6
Molina et al., 2021	[35]	Human	To describe the use of augmented reality (AR)-mediated spine surgery (ARMSS) through a mounted display for guidance to the achieve wide marginal resection of an L1 chordoma.	1 patient	Case study	X Vision, AR-HMD	AR	Osteotomies and discectomies.	En bloc wide marginal resection of an L1 chordoma.	The present AR-HMD platform enabled improved integration of surgical navigation for en bloc spinal tumor resections without minimal line of sight interruption and attention shifts.	JBI Case-study/6
Yahanda et al., 2021	[36]	Human	To assess the accuracy of the percutaneous pedicle screws placed at a single institution using an AR head-mounted display (ARHMD) system.	9 patients	Case series	X Vision, AR-HMD	AR	Pedicle screw placement.	Gertzbein–Robbins scale, thoracic pedicle sub-analysis	AR-guided surgery demonstrated a 100% accuracy rate for the insertion of 63 percutaneous pedicle screws in nine patients.	JBI Case-series/9
Liu et al., 2021	[37]	Human	To report the accuracy of 205 pedicle screws placed using AR assistance with a unique head-mounted display (AR-HMD).	28 patients	RCT	AR-HMD	AR	Pedicle screw placement.	Gertzbein–Robbins scale.	An accuracy rate of 98% was found for AR-assisted pedicle screw placement in 28 patients.	JBI Case-series/9
Zawy Alsofy et al., 2020	[38]	Human	To compare postoperative clinical and radiological complications as well as results of stand-alone cage or cage plate insertion as well as role of VR in that.	107 patients	Case series	3D Slicer platform	VR	Stand-alone cage or cage with plate insertion.	Statistical probability using Fischer’s exact test.	The rate of subsidence, kyphosis, and VAS for neck pain in the group without VR-assisted surgery was higher compared with the one with the VR assisted-surgery. No significant differences were found regarding perioperative complication rates and fusion rates.	JBI Case-series/9
Farshad et al., 2021	[39]	Human	To report the first human case using direct holographic navigation for pedicle screw placement.	1 patient	Case study	AR- based holographic navigation system	AR	Lumbar pedicle screw placement.	Angular deviation of placed screws.	The patient was present with complete reduction in leg pain and showed no further signs of radiculopathy.	JBI Case-study/5
Yoon et al., 2021	[40]	Human	To present a DIY method to build a custom, affordable AR heads-up display system.	3 patients	Case series	DIY AR-HUD	AR	Spinal fusion surgery.	Total operative time.	The custom AR display system was ergonomic and reduced the total operative time compared with traditional methods.	JBI Case-series/6
Aoyama et al., 2021	[41]	Human	To report the utility of the AR-HMD at a lower cost to identify the osteotomy area of a laminectomy for spinal decompression surgery.	2 patients	Case series	AR- HMD	MR	Spinal decompression surgery.	3D CT/MRI fusion images.	Preoperative confirmation of the decompression area could reduce the risk, time, and cost of the surgical procedure.	JBI Case-series/7
Gu et al., 2020	[42]	Human	To compare and study the effects of mixed reality-assisted lumbar pedicle screw placements with traditional screw placement.	50 patients	RCT	HoloLens II with MR system	MR	Lumbar pedicle screw placements.	Intraoperative time, blood loss, success rate of first tapping, number of intraoperative c-arm scans, total postoperative drainage volume.	The group use of mixed reality navigation showed shorter operating times, less bleeding, higher success rates of threading, and less c-arm x-ray irradiation.	JBI RCT/10
Pojskić et al., 2021	[43]	Human	To analyze the use of intraoperative computed tomography with navigation and the implementation of AR in facilitating a lateral approach to spine.	104 patients	Case series	Intraoperative CT	AR	Surgeries with a lateral approach to the spine.	Target mean registration error.	Automatic registration applying intraoperative CT and implementation of AR resulted in higher accuracy (registration error 0.84 mm) with a lower effective radiation dose (4.51 vs. 6.16 mSv).	JBI Case-series/8
Buch et al., 2020	[44]	Human	To construct, visualize, register intraoperative holographic models of patient landmarks during spinal fusion surgery,	7 Patients	Case series	HoloLens	MR	Spinal fusion surgery.	Mean values of accuracy and standard regression.	A custom and accessible low-cost pipeline, which enables a 3D model rendering and registration of the intraoperative imaging obtained by low-resolution, portable CT, improved the mean registration errors from 20.2 mm to 4.18 mm.	JBI Case-series/6
Burström et al., 2021	[45]	Human	To test the hypothesis that intraoperative cone beam computed tomography (CBCT) using the Allura ARSN system in a dedicated hybrid operating room (OR) matches computed tomography (CT) for the identification of pedicle screw breaches during spine surgery.	20 Patients	Case series	Allura ARSN	AR	Spinal fixation surgery.	Grading for the degree of the pedicle screw breach.	The negative predictive value of the intraoperative cone beam computed tomography for a pedicle screw breach was 99.6%, with a sensitivity of 90.0% and a specificity of 97.6%. This result suggests that routine postoperative CT scans are unnecessary.	JBI Case-series/10
Su et al., 2020	[46]	Human	To assess the effectiveness of a new assessment tool, myelopathy-hand functional evaluation system (MFES), in evaluating the hand dysfunction of patients with cervical myelopathy in the 10 s grip-and-release test.	198 Patients	Case series	Myelopathy-hand functional evaluation system (MFES)	VR	Evaluation of cervical myelopathy.	10 second grip and release test (time).	Myelopathy patients had fewer G-R cycles, longer cycle times, and lower/wider ulnar waveforms than healthy patients. MFES can be an objective and quantitative assessment tool for patients suffering from cervical myelopathy.	JBI Case-series/9
Bekelis et al., 2017	[47]	Human	To investigate the effect of exposure to a VR environment preoperatively on patient-reported outcomes for surgical operations.	127 Patients	RCT	Immersive preoperative VR experience	VR	Cranial and spinal surgeries.	EVAN g score, VAS satisfaction and stress score.	Patients exposed to preoperative immersive VR experience showed higher rates of satisfaction during surgical encounters based on EVAN g scores, APAIS scores, preoperative VAS stress scores, VAS preparedness, and VAS satisfaction.	JBI RCT/11
Burström et al., 2020	[48]	Human	To evaluate the accuracy of a new, frameless reference marker system for patient tracking by analyzing the effect of the vertebral position within the surgical field.	4 cadavers and 20 patients	Case series	Adhesive optical skin markers	AR	Pedicle screw placement.	Distance between the planned position and the placed pedicle device.	The AR surgical navigation system with adhesive skin markers had an overall technical accuracy of 1.65 mm and maintained high accuracy throughout the surgical field independent of the vertebral position.	JBI Case-series/9
Charles et al., 2021	[49]	Human	To evaluate the accuracy of the percutaneous pedicle screw placement using augmented reality surgical navigation during minimally invasive transforaminal lumbar interbody fusion (TLIF).	20 Patients	Case series	ARSN	AR	Transforaminal lumbar interbody fusion.	Gertzbein score.	Clinical accuracy using ARSN for screw placement within the pedicle was obtained at 94%.	JBI Case-series/9
DeSalvatore et al., 2020	[50]	Human	To test a novel 3D model created using Google Cardboard for surgical planning for adolescent idiopathic scoliosis patients.	60 patients	Case series	Google Cardboard	VR	AIS correction surgery.	Operative time and bleeding.	Use of this VR-based technology led to decreased operative time and bleeding while increasing surgeon’s satisfaction in a reproducible, cost-effective manner.	JBI Case-series/9
Jamshidi et al., 2021	[51]	Human	To introduce AR-HMD and demonstrate safety and precision while using it for endoscopic transforaminal interbody fusion.	1 patient	Case study	AR-HMD	AR	Endoscopic transforaminal interbody fusion.	Accuracy of endoscopy probe.	Use of AR-HMD enables accurate placement of endoscope to assist discectomy.	JBI Case-study/6
Benjamin et al., 2019	[52]	Human	To present a case series of two patients with ISCN, the first to combine the use of DTI, pre- and intraoperative three-dimensional (3D) virtual reality imaging, and microscope-integrated navigation with heads-up display.	2 patients	Case series	Diffusion tensor imaging (DTI)	VR	ISCN resection.	Confirmation of midline despite abnormal swelling of the cord through the combination of diffusion tractography and MRI.	Use of VR allowed for the real-time visualization of the 3D tractography to assist intramedullary tumor resection.	JBI Case-series/6
Charles et al., 2022	[53]	Human	To assess intra- and inter-observer reliability of pedicle screw placement and to compare the perception of baseline image quality (NoMAR) with optimized image quality (MAR).	24 patients	Case series	MAR and CBCT	AR	Assessment for accuracy of pedicle screw placement.	Gertzbein– Robbins scale	Intraoperative screw positioning can be reliably assessed on cone beam CT for AR surgical navigation when using optimized image quality. MAR and NoMAR images demonstrated good intra-observer and excellent inter-observer and intra-class correlation coefficients.	JBI Case-series/9
Zawy Alsofy et al., 2021	[54]	Human	To analyze patients who underwent MIS or monosegmental dorsal fusion and the compare surgical outcomes along with the complication rates.	171 patients	Case series	DICOM, NIVIDIA GTX1080	VR	MIS or monosegmental dorsal fusion.	Duration of hospital stay, post-surgical complications and recovery time.	MIS was associated with less blood loss, shorter surgery time and hospital stay, lower complication rates, and equivalent long-term patient-reported outcomes, but it was associated with lower fusion rates and higher late reoperation rates than open surgery.	JBI Case-series/9

Abbreviations: AR (augmented reality), ARCASS (AR computer-assisted spine surgery), ARMSS (AR-mediated spine surgery), ARSN (AR surgical navigation), CBCT (cone beam computed tomography), DICOM (digital imaging and communications in medicine), DIY (do it yourself), DTI (diffusion tensor imaging), ECL (extraneous cognitive load), GS (Gertzbein–Robbins scale), HGS (Heary–Gertzbein scale), HMD (head-mounted display), ISCN (intramedullary spinal cord neoplasms), IVRSS (immersive virtual reality surgical simulator), MFES (myelopathy-hand functional evaluation system), MIS (minimally invasive surgery), MR (mixed reality), PSP (pedicle screw placement), RCT (randomized control trial), TLIF (transforaminal lumbar interbody fusion), VAS (visual analogue scale), VR (virtual reality), VSTS (virtual surgical training system).

## Data Availability

No new data were created or analyzed in this study. Data sharing is not applicable to this article.

## Supplementary Materials

The following supporting information can be downloaded at: https://www.mdpi.com/article/10.3390/medicina60020332/s1, File S1: Reporting Guideline: PRISMA Checklist for Scoping Reviews. File S2: Risk of Bias Assessment. Reference [82] is cited in File S1.

## Appendix A

**Table A1:** List of search terms used in two bibliographic databases to identify articles for a systematic review of the role of virtual and augmented reality in trainee education, patient education, surgical execution, and surgery rehearsal, with a focus on spine surgery.

Bibliographic Databases	Search String	Number of Results Obtained	Date the Search Was Run
Scopus	TITLE-ABS-KEY (({Virtual reality} OR {Augmented reality} OR {Mixed reality}) AND ({spine surgery}) AND ((outcome*) OR {execution} OR {education} OR {training} OR {assessment} OR {rehearsal} OR {planning}))	136	23 January 2023
PubMed	(“Virtual reality” OR “Augmented reality” OR “Mixed reality”) AND (“spine surgery”) AND (“outcome*” OR “execution” OR “education” OR “training” OR “assessment” OR “rehearsal” OR “planning”)	92	23 January 2023
